# A smartphone comes to the rescue during tracheostomy

**DOI:** 10.1186/s13054-019-2324-x

**Published:** 2019-01-24

**Authors:** Animesh Ray, Bharath, Sanjeev Sinha, Saurav Sekhar Paul

**Affiliations:** All India Institute of Medical Sciences, New Delhi, India

We had a teenage boy in our ICU, who was a case of acute inflammatory demyelinating polyneuropathy (AIDP) with type 2 respiratory failure and requiring mechanical ventilatory support. In view of prolonged mechanical ventilation, he was taken up for percutaneous tracheostomy (PCT) under bronchoscopic guidance [1].

After taking proper consent, the procedure was started under mild sedation and local instillation of lignocaine. After insertion of the needle into the trachea (confirmed by visualization of needle through bronchoscope), a guide wire was inserted. However, all of a sudden, the light source stopped working during the procedure while the bronchoscope was in situ and the procedure was underway. We were left with two options—either to abort the procedure or to complete the procedure without bronchoscopic guidance. However, the chance of damage to the posterior tracheal wall during dilatation and subsequent tracheostomy tube insertion could not be undermined. Since another light source was not available, we decided to use our smartphone torch to see if it could be used as a substitute. The cable of the bronchoscope that connects to the light source was apposed to the camera flash after turning on the flashlight as shown in the picture (Fig. 1). To our surprise, the smartphone torch provided enough illumination for us to complete the procedure. Dilatation was done with blue rhino dilator and subsequently the tracheostomy tube was inserted under direct visualization.

**Fig. 1:**
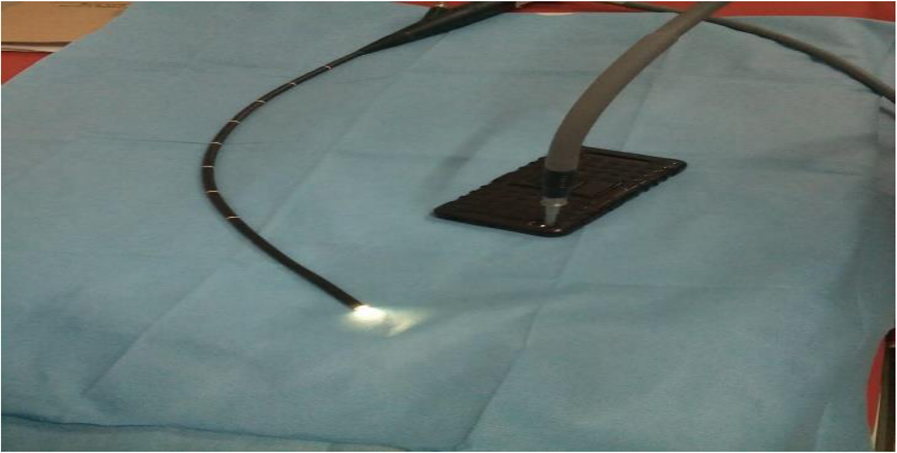
A smartphone torch being used as a light source for a bronchoscope

Thorough literature search afterwards yielded a similar experience being reported previously [2]. Deshmukh et al. had reported the use of smartphone torchlight during a thoracoscopic procedure when their light source had malfunctioned. During malfunctioning of the light source, our “quick fix” was useful and saved the day.

## Conclusion

Though it cannot be routinely advocated, on the backdrop of light source malfunction during a critical procedure like percutaneous tracheostomy (under bronchoscopic guidance), a smartphone can be used as an alternative to provide adequate illumination.
